# Targeted proteomics reveal histiocytosis‐associated neurodegeneration signatures

**DOI:** 10.1002/hem3.70470

**Published:** 2026-09-07

**Authors:** Egle Kvedaraite, Magda Lourda, Johannes Bastiaan Munting, Matthias Papo, Martin Jädersten, Yoko Shioda, Kenichi Sakamoto, Daniel Martín Muñoz, Panagiotis Andriopoulos, Francesco Pegoraro, Evaldas Laurencikas, Ingrid Kühnle, Joakim Wille, Leonie Naeije, Helena Olkinuora, Indranil Sinha, Daniel W. Hagey, Selma Olsson‐Åkefeldt, Fleur Cohen‐Aubart, Gustaf Österlundh, Mattias Svensson, Ahmed Idbaih, Fredrik Piehl, Kaj Blennow, Henrik Zetterberg, Tatiana Greenwood von Bahr, Désirée Gavhed, Akira Morimoto, Julien Haroche, Jan‐Inge Henter

**Affiliations:** ^1^ Department of Pathology and Cancer Diagnostics Karolinska University Hospital Stockholm Sweden; ^2^ Unit of Molecular Neurobiology, Department of Medical Biochemistry and Biophysics, Karolinska Institutet Stockholm Sweden; ^3^ The Systems Virology Laboratory, Department of Laboratory Medicine, Division of Clinical Microbiology, Karolinska Institutet Stockholm Sweden; ^4^ Internal Medicine Department 2, French National Referral Center for Histiocytoses Sorbonne University, Assistance Publique–Hôpitaux de Paris, Pitié‐Salpêtrière Hospital Paris France; ^5^ Department of Medicine Huddinge, Center for Hematology and Regenerative Medicine, Karolinska Institutet Stockholm Sweden; ^6^ Department of Hematology Karolinska University Hospital Stockholm Sweden; ^7^ Children's Cancer Center, National Center for Child health and Development Tokyo Japan; ^8^ Department of Pediatrics Shinshu University School of Medicine Matsumoto Japan; ^9^ Department of Neuroradiology and Pediatric Radiology Karolinska University Hospital Solna Stockholm Sweden; ^10^ Department of Experimental and Clinical Medicine University of Florence Florence Italy; ^11^ Department of Hematology and Oncology, Meyer Children's Hospital IRCCS Florence Italy; ^12^ Childhood Cancer Research Unit, Department of Women's and Children's Health, Karolinska Institutet Stockholm Sweden; ^13^ Division of Pediatric Hematology and Oncology Göttingen University Medical Center Göttingen Germany; ^14^ Childhood Cancer Center Skåne University Hospital Lund Sweden; ^15^ Princess Máxima Center for Pediatric Oncology Utrecht the Netherlands; ^16^ Division of Pediatric Hematology‐Oncology and Stem Cell Transplantation, New Children's Hospital Helsinki University Hospital Helsinki Finland; ^17^ Department of Laboratory Medicine, Division of Biomolecular and Cellular Medicine, Karolinska Institutet Stockholm Sweden; ^18^ Department of Pediatrics, The Queen Silvia Children's Hospital University of Gothenburg Gothenburg Sweden; ^19^ Department of Medicine Huddinge, Center for Infectious Medicine, Karolinska Institutet Karolinska University Hospital Stockholm Sweden; ^20^ Sorbonne Université, AP‐HP, Institut du Cerveau ‐ Paris Brain Institute ‐ ICM, Inserm, CNRS Hôpitaux Universitaires La Pitié Salpêtrière ‐ Charles Foix, Service de Neuro‐Oncologie‐Institut de Neurologie Paris France; ^21^ Department of Neurology Karolinska University Hospital Stockholm Sweden; ^22^ Neuroimmunology Unit, Center for Molecular Medicine, Karolinska Institutet Stockholm Sweden; ^23^ Department of Clinical Neuroscience, Karolinska Institutet Stockholm Sweden; ^24^ Department of Psychiatry and Neurochemistry, Institute of Neuroscience and Physiology, Sahlgrenska Academy University of Gothenburg Mölndal Sweden; ^25^ Clinical Neurochemistry Laboratory Sahlgrenska University Hospital Gothenburg Sweden; ^26^ Institute of Neurology University College London London UK; ^27^ Pediatric Oncology, Astrid Lindgren Children's Hospital Karolinska University Hospital Stockholm Sweden; ^28^ Division of Pediatrics, Showa Inan General Hospital Komagane Japan

## Abstract

Neurodegeneration (ND) is a severe complication of Langerhans cell histiocytosis (LCH), yet its underlying biology and reliable biomarkers remain poorly defined. The aim of this study was to (1) gain insight into neuroimmunological mechanisms governing ND and (2) assess the clinical value of established and novel biomarkers for ND‐LCH. We applied targeted proteomics and neurofilament light chain (NFL) assays to cerebrospinal fluid (CSF) and plasma from LCH patients with and without ND, with control cohorts. ND‐LCH exhibited a distinct CSF proteomic profile characterized by increased decoy receptors, including interleukin‐1 receptor type 2 (IL‐1RT2), macrophage activation markers, and cytotoxic and immunoregulatory proteins. CSF IL‐1RT2 showed higher specificity for ND‐LCH than CSF NFL (0.99 [0.99–1.0] vs. 0.88 [0.80–0.96]) and tracked longitudinal disease changes. Plasma analyses revealed a complementary immune signature in which NFL correlated with CSF markers, including IL‐1RT2, and distinguished ND‐LCH from controls. In independent plasma NFL cohorts from France and Japan, elevated levels were confirmed in adults with histiocytosis‐associated ND. These findings define a neuroinflammatory signature of ND‐LCH and identify IL‐1RT2 as a novel candidate biomarker for diagnosis and monitoring.

## INTRODUCTION

Several histiocytic disorders, including Langerhans cell histiocytosis (LCH), Erdheim‐Chester disease (ECD), and Rosai‐Dorfman‐Destombes disease (RDD), arise from the mononuclear phagocyte lineage.^1^, ^2^, ^3^, ^4^, ^5^ These diseases present with diverse clinical manifestations, including central nervous system (CNS) involvement such as neurodegeneration (ND), which remains a major clinical challenge.^6^, ^7^, ^8^

Among these disorders, LCH is the most common, and it affects both children and adults.^8^, ^9^ LCH is classified as a myeloid inflammatory neoplasia, and patients often respond well to BRAF/MEK inhibitors (BRAFi/MEKi).^10^, ^11^ While survival in LCH now is almost 100% thanks to BRAFi/MEKi, long‐term sequelae remain a major challenge. The broad spectrum of CNS‐LCH includes tumorous lesions and ND, often occurring in combination. The tumorous lesions are most often located in the hypothalamic‐pituitary region and lead to various endocrine disorders, most commonly diabetes insipidus (DI), but can also occur in the pineal gland and other brain parenchymal regions. ND‐LCH is typically located in the cerebellum and basal ganglia, and appears as bilateral, symmetric signal abnormalities on MRI scans. Less frequent sites include the cerebrum and pons. ND‐LCH may manifest years after remission and present as motor and/or cognitive dysfunction.^12^, ^13^ In long‐term studies, clinical signs of ND‐LCH have been reported in 6%–10% of all children with LCH, and radiologic signs of ND‐LCH are even more frequent.^14^, ^15^, ^16^ Early detection and effective treatment of ND‐LCH remain significant challenges, although there are reports that some patients may respond, at least partly, to BRAFi/MEKi.^17^, ^18^, ^19^, ^20^

Previous research has proposed potential biomarkers for the detection of ND‐LCH, such as osteopontin (OPN) and neurofilament light chain protein (NFL).^20^, ^21^, ^22^, ^23^ NFL in cerebrospinal fluid (CSF) has been suggested as a biomarker for monitoring of ND‐LCH treatment, such as with BRAFi/MEKi, while plasma NFL has been proposed as a candidate for screening of ND in pediatric LCH patients.^24^ Despite these promising findings, unbiased approaches capable of detecting a wide range of soluble proteins remain scarce, and the rarity of histiocytic disorders limits the availability of sufficiently large sample sizes for robust analyses. Moreover, elevated plasma NFL has been reported in a single study in adults with CNS histiocytic neoplasms,^25^ and we aimed to confirm these findings.

The aim of this study was to (1) gain insight into neuroimmunological mechanisms governing ND and (2) assess the clinical value of established and novel biomarkers for ND‐LCH. We applied targeted proteomics in CSF and plasma from LCH patients and multiple control cohorts, combined with sensitive NFL quantification using Simoa. The study included LCH cohorts as well as inflammatory and non‐inflammatory controls, and, in addition, plasma NFL validation cohorts from France and Japan. Finally, we assessed a novel targeted proteomics candidate, interleukin‐1 receptor type 2 (IL‐1RT2), for diagnosis and monitoring of treatment response.

## METHODS

### Patient cohorts and samples

CSF and plasma samples were collected from LCH patients treated or consulted at Karolinska University Hospital, Stockholm, during a period of 12 years, starting in 2007. The discovery cohort included 46 CSF and 23 plasma samples from 30 LCH patients (29 pediatric and one adult; aged 0.6–23.1 years, mean 9.3 years), all of whom had undergone MRI, although data on gadolinium enhancement or brain volume loss were not systematically collected. ND was defined based on neuroradiological evidence of CNS ND (*n* = 10), and several patients had corresponding clinical features (clinical details in Table 1 and Supporting Information S1: Methods). CNS‐LCH was defined as parenchymal CNS involvement (including the hypothalamic‐pituitary region but excluding isolated dural disease). LCH patients without neuroradiological or clinical signs of ND‐LCH served as the non‐neurodegenerative comparison group for both CSF and plasma analyses.

**Table 1:** Clinical characteristics of the discovery LCH cohort.

Patient number	Sample type	ND‐LCH^a^	CNS‐LCH mass lesion^a^	Age^a^	Disease activity at sampling^a^	Ongoing therapy	Neuroradiological findings, areas^b^	Endocrine dysfunction^b^	Neurological/cognitive deficits^b^	Age at diagnosis/Sex	Maximal extent^b^	CNS‐risk lesions^c^	Molecular findings
1	CSF	Yes	Yes	7.9	AD	CS, 6‐MP, VBL, DAC	ND, cerebellum, basal ganglia, brainstem, hypothalamus, hippocampus	DI, GH	Tremor, dysarthria, dysphagia, ataxia, learning disabilities, behavioral abnormalities, gross motor dysfunction	0.5/F	MS ROI+	No	NA
1	CSF	Yes	Yes	8.3	AD	CS, 6‐MP, VBL, MTX, AP							
1	CSF	Yes	Yes	8.7	AD	CS, 6‐MP, MTX, AP							
1	CSF	Yes	Yes	9.1	AD	CS, AP							
1	CSF	Yes	Yes	10.2	AD	Ara‐C, VBL							
1	CSF	Yes	Yes	10.7	AD	AP							
1	CSF	Yes	Yes	12.2	AD	AP, NTZ							
1	CSF	Yes	Yes	12.8	AD	AP							
1	CSF, P	Yes	Yes	14.6	AD	AP							
1	CSF	Yes	Yes	16.1	NAD	CS							
1	CSF	Yes	Yes	16.7	NAD	None							
2	CSF	Yes	Yes	10.2	NAD	None	ND, cerebellum	None	Learning disabilities	1.3/M	MS ROI‐	Yes	NA
3	CSF	Yes	Yes	12.7	NAD	None	ND, basal ganglia, pituitary	DI, GH	Dysarthria, ataxia, learning disabilities, behavioral abnormalities	2.2/M	MS ROI‐	No	NA
4	CSF	NA	Yes	9.8	NA	NA	Spinal cord	None	None	NA/M	NA	NA	NA
5	CSF	Yes	Yes	6.5	AD	None	ND, thalamus, cerebellum, pons, basal ganglia, pituitary	DI, GH	Difficulties in fine motor skills and visual‐spatial perception	5.3/M	SS ROI‐	No	NA
5	CSF	Yes	Yes	8.2	NAD	NA							
6	CSF	No	No	2.4	AD	CS, VBL	None	None	None	2.4/F	MS ROI‐	No	NA
7	CSF	Yes	No	7.2	AD	None	ND, cerebellum, posterior brainstem	DI	Reduced attention in verbal and visual reasoning, general fatigue	6.1/M	SS uf ROI‐	No	NA
7	CSF	Yes	NA	7.6	AD	IVIG							
7	CSF	Yes	No	8.2	AD	NA							
7	CSF	Yes	No	8.6	AD	IVIG							
8	CSF	No	No	5.7	AD	6‐MP, MTX	None	None	None	3.5/M	SS mf ROI‐	No	*BRAF* ^V600E^
9	CSF	Yes	Yes	16	NA	IVIG, MTX, 6‐MP	ND, nuclei dentati, cerebellum white matter, pituitary, corpus pineale	DI	Learning disabilities, balance problems	1.3/M	MS ROI‐	Yes	NA
9	CSF	Yes	Yes	17	NA	IVIG, MTX, 6‐MP							
10	CSF	No	No	10.3	NAD	None	None	None	None	0.2/F	MS ROI+	No	NA
11	CSF	No	No	2.5	AD	None	None	None	None	2.5/M	SS mf ROI‐	Yes	*BRAF* ^V600E^‐ negative
11	CSF, P	No	No	5.6	NAD	None							
12	CSF	Yes	Yes	11.5	AD	2‐CdA, Ara‐C	ND, mesencephalon, pons, pedunculi cerebelli, pituitary	DI, GH	None	2.3/M	MS ROI‐	Yes	*BRAF* ^V600E^‐ negative
13	CSF	No	Yes	6.1	NAD	MTX, 6‐MP	Pituitary	DI	Fluctuating working memory	3/F	MS ROI‐	Yes	NA
14	CSF	No	No	6.2	AD	None	None	None	None	1.8/F	SS mf ROI‐	Yes	NA
15	CSF	No	Yes	9.4	NA	None	Pituitary	DI	Fatigue	2.7/M	SS uf ROI‐	No	*BRAF* ^V600E^
16	CSF, P	No	No	11.1	NAD	None	None	None	None	0.7/M	MS ROI+	Yes	*MAP3K1*
17	CSF, P	No	No	13.3	NAD	None	None	None	None	1.3/F	MS ROI+	Yes	NA
18	CSF, P	No	Yes	8.9	AD	None	Pituitary	DI	None None	8.9/F	SS uf ROI‐	No	NA
18	CSF, P	No	Yes	10	AD	None							
19	CSF, P	Yes	Yes	9.1	AD	None	ND, cerebral and cortical atrophy, hypothalamus, pons, brain stem, basal ganglia, globus pallidus, amygdala, pituitary	DI, GH	Learning disabilities, mental retardation detected by neurophysiological testing	3.5/M	MS ROI‐	No	No mutation detected, but pERK‐positive
19	CSF, P	Yes	Yes	10	AD	CS, Ara‐C, VCR							
19	CSF, P	Yes	Yes	10.4	AD	2‐CdA							
19	CSF, P	Yes	Yes	12	AD	6‐MP, MTX							
20	CSF, P	Yes	Yes	3.2	AD	CS, 6‐MP, MTX	ND, nuclei dentati	DI	Ataxia, learning disabilities	0.4/M	MS ROI‐	Yes	*BRAF* ^V600E^
20	CSF, P	Yes	Yes	4.2	AD	CS, Ara‐C							
21	CSF, P	No	No	2.9	AD	LSI	None	None	None	2.8/M	SS mf ROI‐	Yes	*BRAF* ^V600E^
22	CSF, P	No	No	0.6	AD	None	None	None	None	0.6/F	SS mf ROI‐	Yes	*BRAF* ^V600E^‐ negative
23	CSF	Yes	Yes	23.1	AD	None	ND, pons, cerebellum, vermis, nuclei dentati, hypothalamus	DI, HyperNa, ED, AM	Tremor, ataxia, atypical anorexia nervosa, intermittent headache, wheelchair user	23/F	MS ROI‐	No	*BRAF* ^V600E^
23	P	Yes	Yes	22.9	AD	None							
23	P	Yes	Yes	23	AD	None							
24	CSF, P	No	No	0.7	AD	None	None	None	None	0.6/F	MS ROI‐	Yes	*BRAF* ^V600D^
25	CSF, P	No	No	1.6	AD	None	None	None	None	1.5/F	MS ROI‐	Yes	*BRAF* ^V600E^
26	P	No	No	6.4	AD	None	None	None	None	6.3/M	SS uf ROI‐	No	*BRAF* ^V600E^
27	P	No	No	5.1	AD	None	None	None	None	5.1/M	SS uf ROI‐	No	NA
28	P	No	No	12.3	AD	None	None	None	None	12.1/M	SS mf ROI‐	Yes	*BRAF* ^V600E^‐negative (also pERK negative)
29	P	No	No	2.8	AD	None	None	None	None	2.4/M	MS ROI‐	Yes	*BRAF* ^V600E^
30	P	No	No	2.7	AD	None	None	None	None	2.7/M	MS ROI‐	Yes	*BRAF* ^V600E^

*Note*: Each row represents a sampling occasion, listed in descending chronological order. Age is reported in years.

Abbreviations: 2‐CdA, 2‐chlorodeoxyadenosine (Cladribine); AD, active disease; AM, amenorrhea; AP, apheresis; Ara‐C, cytosine arabinoside (Cytarabine); CS, systemic steroids; CSF, cerebrospinal fluid; CNS, central nervous system; DAC, daclizumab (Zenapax); DI, diabetes insipidus; ED, estrogen deficiency; F, female; GH; growth hormone deficiency; HyperNa, hypernatremia; IVIG, intravenous immunoglobulin therapy; LSI, local steroid injection; mf, multifocal; M, male; MS, multisystem; MTX, methotrexate; NAD, no active disease; ND, neurodegeneration; ND‐LCH, neurodegenerative Langerhans cell histiocytosis; NA, data not available; NTZ, natalizumab; P, plasma; RO +, with risk organ (liver, spleen, hematopoietic system) involvement; RO‐, without risk organ involvement; SS, single system; uf, unifocal; VBL, vinblastine; VCR, vincristine.

a At sampling.

^b^ At max CNS‐LCH until the last sample analyzed.

c CNS‐risk lesions since diagnosis: craniofacial bones (cranial fossa, paranasal sinuses, ethmoid, maxilla, zygomatic, orbit, sphenoid, mastoid, temporal bone).

To strengthen the normal CSF reference group, given the practical and ethical difficulties of obtaining CSF from healthy children, we included 30 anonymized pediatric ALL patients without CNS involvement. For plasma, non‐CNS‐LCH patients served as non‐neurodegenerative references and were complemented by six healthy children recruited at the Pediatric Day Surgery Unit (33% female, ages 3–10 years, mean 5.7 years). Additionally, 12 healthy young adults (50% female, ages 24–30 years, mean 26.2 years) from whom both CSF and plasma were collected were included, along with CSF and plasma samples from patients with multiple sclerosis (MS; 50% female, ages 22–29 years)—a disease with both inflammatory and neurodegenerative components. The MS control cohort consisted of patients with relapsing‐remitting MS (RRMS); half were sampled during remission and half during relapse.

ND‐LCH and non‐ND samples within each fluid compartment were processed in the same laboratories, and young healthy controls and MS controls were processed together to minimize technical batch effects. Prospective validation cohorts were collected in France and Japan. Full details on all patient and control cohorts and samples are provided in Table 1, Supporting Information S1: Tables S1 and S2, and in the Methods of Supporting Information. The study was approved by Ethical Review Committees in Sweden, France, and Japan, as detailed in Supporting Information.

### Sampling and quantification of soluble factors

CSF and plasma were sampled using standard procedures, described in detail in the Methods of Supporting Information. Targeted proteomics (Olink AB, Uppsala, Sweden) was performed in all samples using Immuno‐Oncology (v.3111) and Cardiovascular III (v.6113) panels. After quality control, 177 proteins were analyzed; values below the limit of detection were retained as reported in the Olink NPX output without imputation. NFL levels in CSF were measured using an in‐house ELISA with a normal reference value of <380 ng/L.^26^ As plasma NFL levels are substantially lower than in CSF, plasma NFL levels were quantified using the more sensitive Quanterix Simoa NFL assay, which has a dynamic range of 1.9–1812 pg/mL. Age‐specific normal reference values for plasma NFL are as follows: 7 pg/mL for individuals aged 5–<18 years, <10 pg/mL for 18–<51 years, <15 pg/mL for 51–<61 years, <20 pg/mL for 61–70 years, and <35 pg/mL for individuals aged 70 years or older.^27^ Results were adjusted to account for sample dilutions. All NFL analyses were conducted at the Clinical Neurochemistry Laboratory, Sahlgrenska University Hospital, Mölndal, Sweden. For the validation using ELISA, IL‐1RT2 levels in CSF were measured at the Karolinska Institute using the human IL‐1RT2 solid‐phase sandwich ELISA from Invitrogen (cat #: EHIL1R2).

Statistical analyses across multiple groups were performed using the Kruskal–Wallis test with Dunn's post hoc multiple comparisons test, and between two groups using the Mann–Whitney test (Figures 2, 3, 4), and a mixed‐effects model with subject as a random effect was used to compare patients and controls (Figure 4C) using GraphPad Prism (version 10.0.3). Unless otherwise specified, all additional analyses were conducted in R (v4.3.0) using standard base functions and the following packages: dplyr, ComplexHeatmap, RColorBrewer, circlize, corrplot, ggplot2, and pheatmap. See Supporting Information for analytical details.

## RESULTS

### Discovery cohort, control groups, and marker selection for targeted proteomics

**Figure 1:**
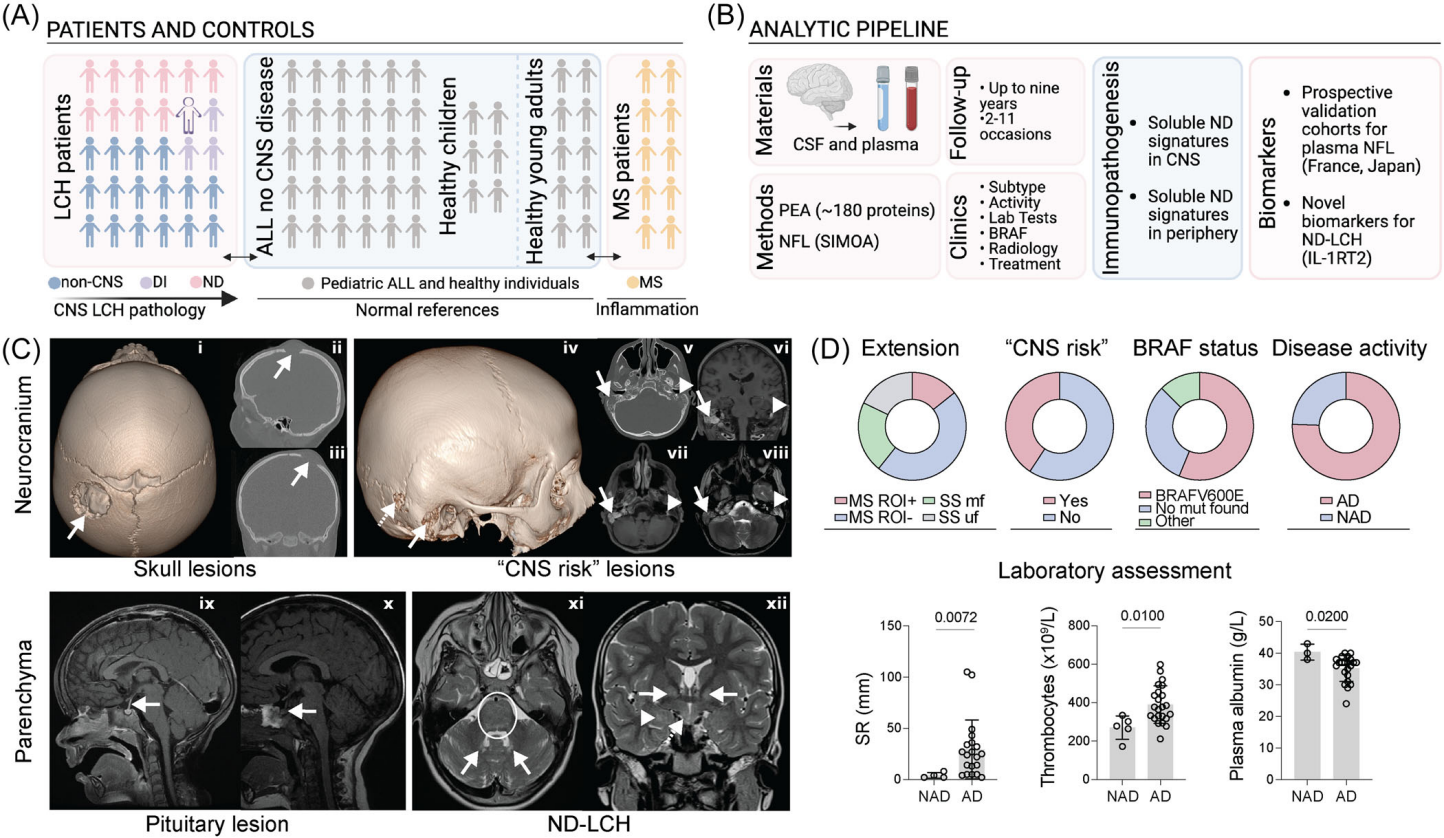
**Study cohorts, design, and clinical characteristics. (A)** Study cohorts: Patients with Langerhans cell histiocytosis (LCH) (*n* = 30) classified by central nervous system (CNS) involvement: no CNS pathology (non‐CNS, *n* = 16), diabetes insipidus (DI, *n* = 3), neurodegenerative disease (ND, *n* = 10), and one with a spinal cord lesion and unknown ND status (indicated by a lilac outline only, *n* = 1). Controls included pediatric ALL patients at diagnosis (no CNS disease, *n* = 30), healthy pediatric controls (HPED, *n* = 6), healthy young adults (HC, *n* = 12), and inflammation controls (young adults with MS, *n* = 12). **(B)** Study design: Overview of materials and methods. **(C)** Neuroradiological findings: Representative imaging of LCH skull lesions shown in CT skull 3D superior posterior view (i), sagittal (ii), and coronal (iii) in bone window with bone defect (arrows); CNS‐risk lesions shown in CT skull 3D right lateral view (iv), axial CT bone window (v), MRI contrast enhanced T1 coronal (vi) and axial T2 (viii) with bone defect in asterion (dotted arrow in iv) and mastoid bone with enhancing soft tissue mass (arrows in iv–viii) and a smaller similar lesion in left mastoid bone (arrow heads in v–viii) eroding the external auditory canal and anterior wall of the sigmoid venous sinus; on T2 lesions have lower signal than the opacified mastoid air cells; hypothalamic‐pituitary involvement shown in MRI sagittal T1 with (ix) and without (x) contrast enhancement with at presentation thick infundibulum (ix) and no neuropituitary (x); ND‐LCH shown in MRI T2 axial (xi) and coronal (xii) with focal lesions in cerebellar nuclei (arrows in xi) and signs of very early volume loss with rounded fastigium of fourth ventricle as well as focal and diffuse lesions in the enlarged pons (circle in xi), lesions in globus pallidi (arrows in xii), right amygdala (arrow head), and hypothalamus (dotted arrow). **(D)** Available clinical characteristics on samples from the discovery LCH cohort; for more details see Table 1. Statistical significance was assessed using the Mann–Whitney test; mean with SD and P‐values shown.

The discovery cohort included CSF and plasma samples from LCH patients with neurodegenerative LCH (ND‐LCH), CNS‐LCH without ND, non‐CNS‐LCH patients with no CNS involvement, and a patient with a spinal cord lesion (Figure 1A). Identifying ND‐associated signatures and novel biomarkers in LCH requires robust quantification of normal references in CSF. However, obtaining CSF samples from healthy individuals is particularly challenging in pediatric and young adult populations. Since CSF sampling in the LCH cohorts was performed based on clinical indications, this inherently introduced a bias toward ND, even among CNS‐LCH without ND and non‐CNS‐LCH patients. While these groups were critical as non‐ND references to control for ND‐LCH, the inclusion of pediatric ALL patients (untreated at diagnosis and without CNS involvement) and healthy young adults further strengthened the non‐ND reference dataset (Figure 1A). Plasma from healthy young children served as an additional non‐ND plasma control. To account for general inflammatory components unrelated to LCH‐associated ND, CSF and plasma samples from MS patients were included as an inflammatory control group (Figure 1A).

For targeted proteomics, the Immuno‐Oncology and Cardiovascular III panels from Olink were selected, and a final total of 177 Olink analytes and NFL (Figure 1B) were investigated in all samples (see Methods). The discovery cohort included samples from 30 LCH patients; neuroradiological findings are illustrated in Figure 1C, clinical data are provided in Table 1, and patient cohorts and samples are described in Methods, Supporting Information, as well as in Figure 1D.

### Targeted proteomics of CSF reveals decoy receptors, macrophage activation, and cytotoxic immune response in ND‐LCH

**Figure 2:**
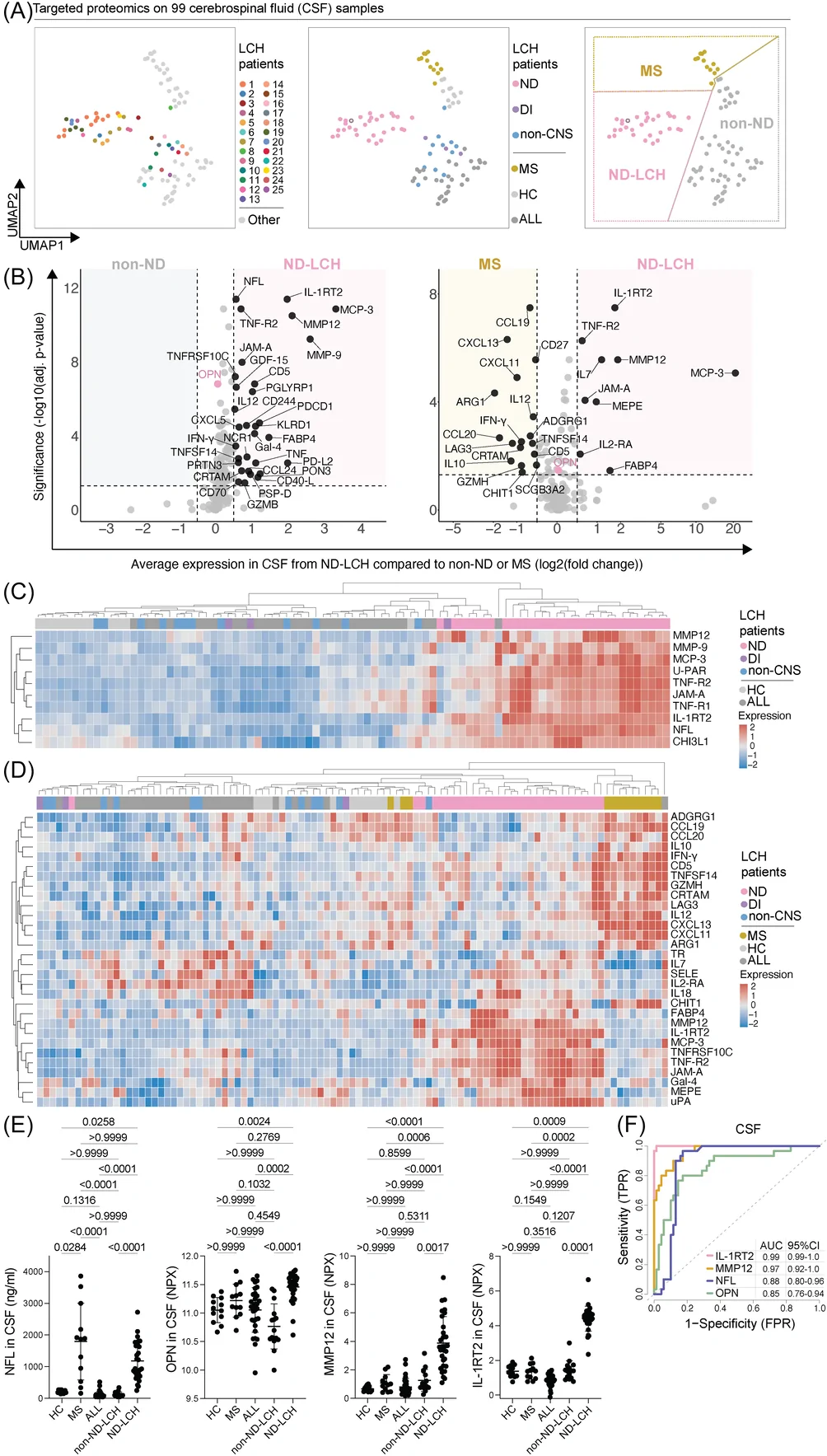
**Targeted proteomics of cerebrospinal fluid (CSF) uncovers novel neurodegeneration markers in Langerhans cell histiocytosis (LCH). (A)** UMAP representation of cerebrospinal fluid (CSF) protein marker expression. Each dot represents a sample from LCH patients (left panel): non‐CNS (*n* = 16), diabetes insipidus (DI) (*n* = 3), neurodegenerative disease (ND) (*n* = 10), and one patient with a spinal cord lesion and unknown ND status (open circle, *n* = 1) or controls: ALL patients at diagnosis with no CNS disease (ALL, *n* = 30), healthy young adults (HC, *n* = 12), and young adults with multiple sclerosis (MS, n = 12) (middle panel). Three major groups are highlighted: the ND‐LCH cluster (pink), non‐ND controls (gray; including DI, non‐CNS, HC, ALL), and inflammation controls (yellow, MS) (right panel). **(B)** Volcano plots comparing average CSF protein expression in ND‐LCH versus non‐ND or MS. The *x*‐axis for the MS comparison uses a non‐linear transformation to enhance the visualization of fold changes across a wide range of values, as indicated by the *x*‐axis scale. Statistical significance was assessed using the Wilcoxon test with FDR‐adjusted P‐values. The dashed line indicates the statistical significance threshold at FDR‐adjusted P‐value < 0.05 and fold change ≥ 1.5. Osteopontin (OPN) is highlighted in pink. **(C)** Heatmap and hierarchical clustering of the top 10 most significant proteins discriminating ND‐LCH from non‐ND. **(D)** Heatmap and hierarchical clustering of the top 30 proteins discriminating ND‐LCH from MS, all 99 samples displayed. **(E)** CSF levels of selected markers compared between the groups were analyzed using the Kruskal‐Wallis test, performed independently for each marker (all P < 0.0001), followed by Dunn's multiple comparisons test. Data are presented as mean with SD, with all adjusted P‐values indicated. **(F)** Receiver operating characteristic (ROC) curves for IL‐1RT2, MMP12, OPN, and NFL in CSF samples comparing ND‐LCH versus all other CSF samples; the false positive rate (FPR, 1 − specificity) on *x*‐axis, and the true positive rate (TPR, sensitivity) on *y*‐axis; area under the curve (AUC) values shown. All comparisons are performed between the groups shown in the right panel of A: ND‐LCH (*n* = 11), non‐ND controls (*n* = 61), and MS controls (*n* = 12).

Using soluble protein data, the CSF samples from ND‐LCH patients and from a patient with a spinal cord lesion (ND status for this patient was unknown) formed a distinct cluster in UMAP space, separating from both MS patients and non‐ND controls. The CSF proteomic profile of the spinal cord lesion patient clustered with those of ND‐LCH patients (Figure 2A), and the sample was included in the ND‐LCH cluster for all analyses. The non‐ND controls included CNS‐LCH patients without ND, LCH patients without CNS involvement, ALL patients, and healthy young adults (Figure 2A). Differential expression analysis identified 98 proteins differing between ND‐LCH and non‐ND controls. Proteins upregulated in ND‐LCH were enriched for immune response markers, including macrophage activation and remodeling (e.g., CD163, CHIT1, MMP12, and OPN) and cytotoxic immune responses (e.g., granzymes, perforin, and NK cell markers) (Figure 2B, left). Markers of T‐cell co‐stimulation (e.g., CD70, TNFSF14, and CD40‐L) and immune regulation (e.g., CD5, PDCD1, which is PD1) were also elevated, indicating adaptive immune involvement. Consistent with ND, NFL and GDF‐15 were upregulated, supporting ongoing neurodegenerative processes alongside complex innate and adaptive immune activation.

The most elevated proteins in ND‐LCH versus non‐ND were IL‐1 receptor type 2 (IL‐1RT2), the monocyte chemoattractant MCP‐3/CCL7, and the monocyte/macrophage metalloprotease MMP12 (Figure 2B, left). These markers, together with TNF receptor 2 (TNFR2), remained distinctly elevated in ND‐LCH compared with MS (Figure 2B, right). In contrast, lymphocyte‐associated proteins involved in immune activation, survival, and migration—including CXCL13, CCL19, CXCL11, IL‐12, IFN‐γ, CCL20, CRTAM, and LAG3—were higher in MS than in ND‐LCH.

Based on the top 10 DEPs distinguishing ND‐LCH from non‐ND, most ND‐LCH samples clustered together (Figure 2C). Hierarchical clustering using the top 30 DEPs separating ND‐LCH from MS further revealed distinct ND‐LCH, MS, and non‐ND clusters (Figure 2D). Analysis of established (NFL and OPN) and novel (IL‐1RT2 and MMP12) ND‐LCH markers showed significant group differences (Figure 2E). NFL and OPN levels did not differ between ND‐LCH and MS, suggesting shared neurodegenerative or inflammatory features, whereas IL‐1RT2 and MMP12 were specific to ND‐LCH, even relative to MS. Notably, IL‐1RT2 showed the strongest discriminative performance, with the highest receiver operating characteristic area under the curve (ROC AUC) of 0.99 (95% confidence interval [CI], 0.99–1.0) (Figure 2F). This performance remained unchanged after exclusion of the spinal cord patient who clustered with ND patients but had an unknown ND status.

These findings suggest a unique CSF protein signature in ND‐LCH, characterized by macrophage activation, inflammatory cytokine production, and extracellular matrix remodeling, all of which may contribute to disease pathogenesis and ND. Additionally, novel ND‐LCH marker candidates, such as IL‐1RT2 and MMP12, were identified.

### ND‐LCH plasma proteomic signatures reveal ND and CNS‐enriched immune alterations

**Figure 3:**
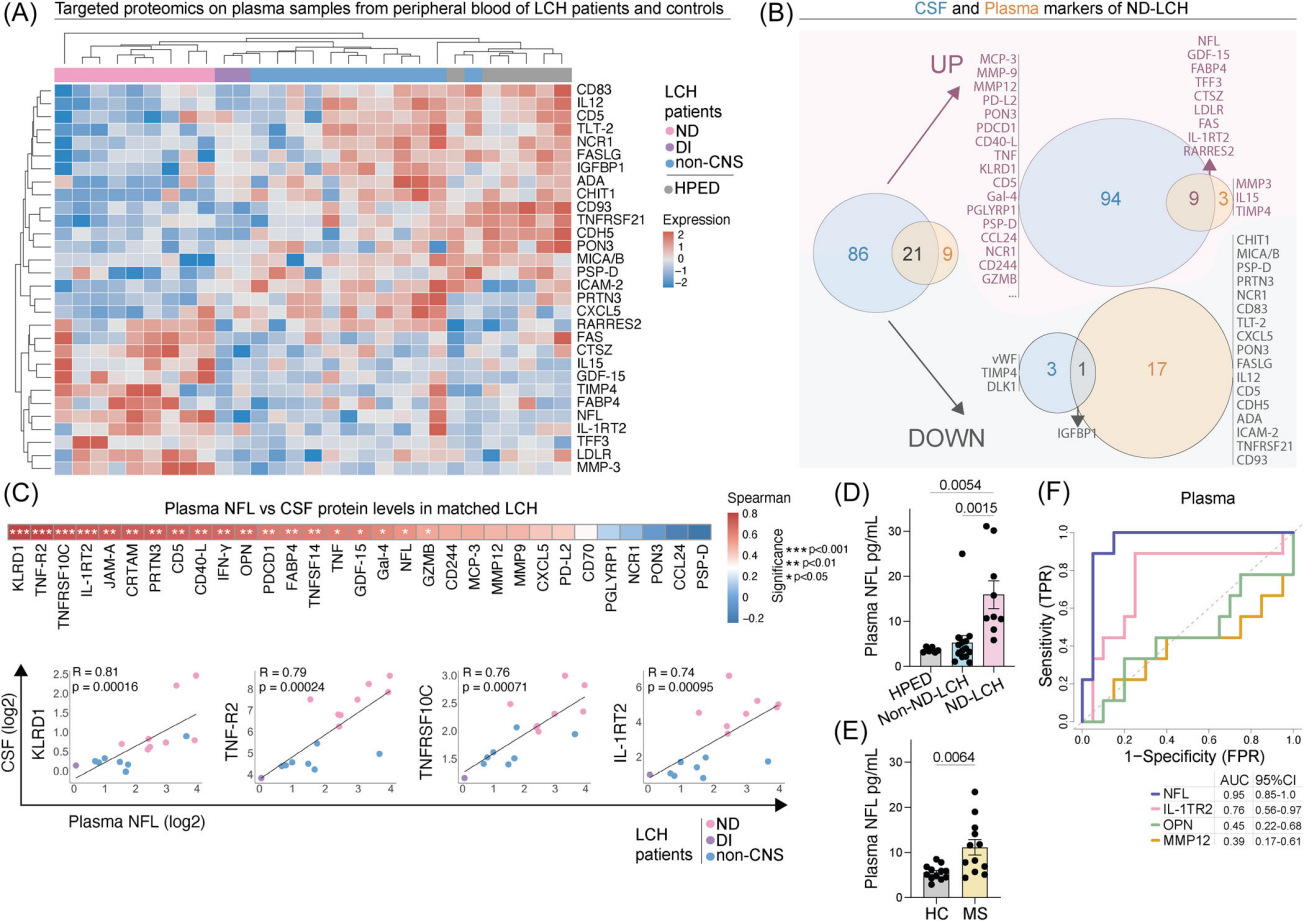
**Targeted proteomics of plasma from peripheral blood suggests neurodegeneration‐related markers in Langerhans cell histiocytosis (LCH). (A)** Heatmap and hierarchical clustering of proteins with significant differential expression (P < 0.05) distinguishing neurodegeneration (ND)‐LCH (*n* = 4) from non‐ND (*n* = 19) in plasma. **(B)** Venn diagrams showing the overlap of differentially expressed proteins (P < 0.05) between plasma (orange filled circles) and CSF (blue filled circles) in ND (*n* = 4 for plasma, *n* = 11 for CSF) versus non‐ND (*n* = 19 for plasma, *n* = 61 for CSF), with upregulated proteins shown in dark purple (upper right) and downregulated proteins shown in dark gray (lower right). **(C)** Spearman correlation heatmap showing plasma neurofilament light chain (NFL) and top 30 cerebrospinal fluid (CSF) ND‐LCH markers (based on fold change) plus OPN in 16 matched LCH samples (P‐values: *P < 0.05, **P < 0.01, ***P < 0.001), with scatter plots below depicting plasma NFL (log2) versus CSF marker levels, and a black line indicating linear regression fit. **(D)** Plasma NFL levels in healthy pediatric controls (HPED, *n* = 6), LCH patients without ND (non‐ND‐LCH, *n* = 14), and with ND (ND‐LCH, *n* = 4), analyzed via Kruskal–Wallis (P‐value 0.0006), followed by Dunn's multiple comparisons test. Data are presented as mean with SEM, with adjusted P‐values indicated. **(E)** Plasma NFL levels in healthy young adults (HC, *n* = 12) and MS patients (*n* = 12), analyzed via two‐tailed Mann–Whitney test (P‐values and mean with SEM shown). **(F)** ROC curves for IL‐1RT2, MMP12, OPN, and NFL in plasma samples comparing ND‐LCH (*n* = 4) versus non‐ND (DI, non‐CNS, HPED, *n* = 19); the false positive rate (FPR, 1 − specificity) on *x*‐axis, and the true positive rate (TPR, sensitivity) on *y*‐axis; area under the curve (AUC) values shown.

Hierarchical clustering of plasma samples from LCH patients and pediatric controls revealed distinct clustering patterns (Figure 3A). Notably, ND‐LCH samples formed a separate cluster, even at the plasma level, distinguishing them from CNS‐LCH samples with DI but without ND, as well as from LCH without CNS involvement. Moreover, while both non‐CNS LCH patients and pediatric controls were distinct from ND‐LCH and CNS‐LCH samples overall, most samples from these two groups also showed separation from each other (Figure 3A). Key proteins differentiating ND‐LCH from non‐ND patients included NFL and MMP‐3, a metalloprotease capable of activating other metalloproteases. In contrast, the control group displayed relatively higher levels of immune activation and cell‐interaction markers (e.g., IL‐12, NCR1, TLT2, CD83, CHIT1, and MICA/B), suggesting their relative depletion in ND‐LCH plasma.

As expected, we did not observe complete concordance between CSF and plasma, and we next compared DEPs in CSF and plasma samples to reveal compartment‐specific proteomic characteristics (Figure 3B). Overall, most proteins elevated in ND‐LCH were also found to be upregulated in the CSF, while most of the downregulated proteins showed decreased levels in plasma. Interestingly, six proteins, that is, IL‐12, NCR1, CHIT1, PSP‐D, PRTN3, and CD93 (adjusted FDR P‐value < 0.05), were significantly upregulated in CSF and downregulated in plasma, indicating a compartment‐specific distribution pattern consistent with preferential enrichment within the CNS compartment in ND‐LCH.

Next, we assessed how plasma NFL, previously suggested as a biomarker for CNS‐LCH, correlated with the top 30 CSF ND‐LCH markers, as well as OPN in CSF, using matched CSF and plasma samples from LCH patients (Figure 3C). This analysis revealed the strongest correlations between plasma NFL and KLRD1, TNF‐R2 (TNFRSF1B), TNFRSF10C, and IL1RT2 (Figure 3C).

Finally, we confirmed elevated plasma NFL levels in ND‐LCH patients (predominantly pediatric) compared to non‐ND‐LCH and pediatric controls (Figure 3D). This elevation was not specific to LCH, as MS patients also exhibited increased plasma NFL levels relative to age‐matched controls (Figure 3E). In ROC analyses, plasma NFL outperformed other plasma ND‐LCH markers, as well as other CSF ND‐LCH markers including OPN when measured in plasma (Figure 3F).

Collectively, these findings show that ND‐LCH patients exhibit a distinctive proteomic signature that is compartment‐specific, characterized by reduced levels of several immune activation markers that are enriched in CSF and by a strong correlation between plasma NFL and the ND‐LCH CSF proteomic pattern.

### Plasma NFL is elevated in adults with histiocytosis‐associated ND, including patients on BRAF/MEK inhibitors

Given that plasma NFL showed the strongest signal among candidate plasma markers in the predominantly pediatric discovery cohort (Figure 3), we focused subsequent analyses on its clinical relevance in adults.

**Figure 4:**
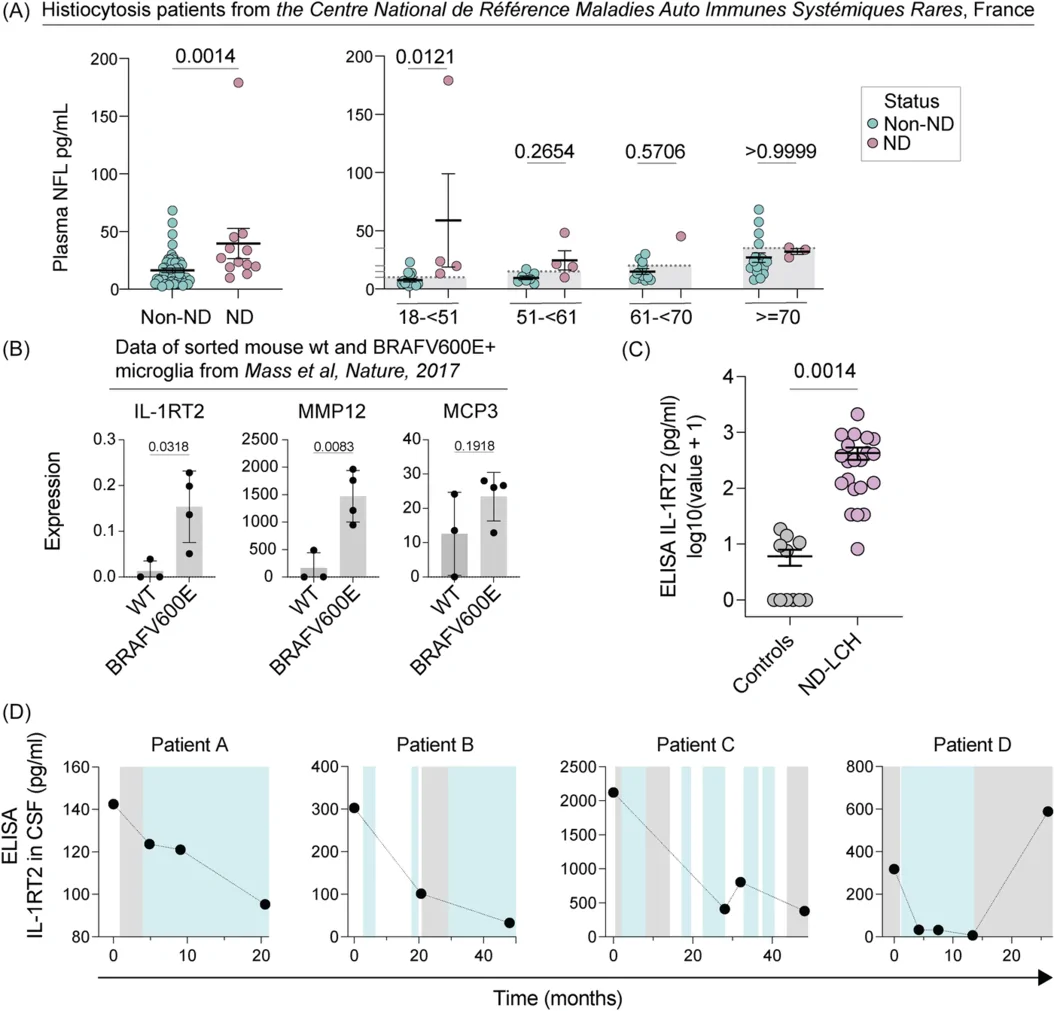
**Validation of plasma neurofilament light chain (NFL) in a prospective cohort from France and evaluation of interleukin‐1 receptor type 2 (IL‐1RT2) as a novel diagnostic and therapy response candidate biomarker. (A)** Plasma NFL levels in histiocytosis patients (*n* = 59) with and without neurodegeneration from the French cohort, analyzed using a two‐tailed Mann–Whitney test (left). Age‐stratified data are shown on the right and analyzed using the Kruskal–Wallis test (P‐value < 0.0001), followed by Dunn's multiple‐comparisons test. The normal reference range is shown in gray. All adjusted P‐values are indicated, and mean with SEM shown. **(B)** Expression level of IL‐1RT2, MMP12, and MCP‐3 between mouse microglia with and without *BRAF* ^V600E^; *in vivo* data re‐used from Mass et al.^28^; analyzed via unpaired *t*‐test (all P‐values indicated and mean with SD shown). **(C)** IL‐1RT2 ELISA in cerebrospinal fluid (CSF) of 11 controls sampled once (ALL patients without central nervous system (CNS) involvement) and five neurodegeneration (ND)‐Langerhans cell histiocytosis (LCH) patients sampled four to five times (see Methods in Supporting Information); analyzed using a mixed‐effects model (REML) (P‐value and mean with SEM shown). **(D)** IL‐1RT2 ELISA in CSF over time (months) in four ND‐LCH patients treated with BRAF/MEK inhibitors, starting from the first pre‐treatment/off‐treatment sample if available and subsequent samples; light green indicates BRAF/MEKi treatment, gray indicates other treatment, and white is untreated (for detailed treatment descriptions see Methods in Supporting Information).

To this end, we analyzed a prospective French cohort of 59 adults with histiocytosis (Figure 4A, for clinical details, see Supporting Information S1: Table S1). The French cohort included 59 adults diagnosed with LCH, ECD, RDD, or mixed‐type histiocytosis, 10 of whom had ND (Supporting Information S1: Table S1). All ND patients harbored the *BRAF* ^V600E^ mutation and were receiving treatment at the time of sampling: nine were treated with BRAF and/or MEK inhibitors (BRAF/MEKi), while one was on Pegylated Interferon‐alpha (Supporting Information S1: Table S1). In this cohort, plasma NFL levels were higher in ND patients compared with non‐ND patients (median 25.5 pg/mL, IQR 19.3–42.8 vs. median 13.1 pg/mL, IQR 7.2–23.2) (Figure 4A). NFL levels also increased with age, consistent with known age dependency of NFL levels (Figure 4A).

We also evaluated an independent Japanese cohort (Supporting Information S1: Table S2 and Supporting Information S1: Figure S1), which was not powered for formal inference due to limited sample size and heterogeneity. Of note, two patients in the non‐CNS group of this cohort had elevated plasma NFL levels; both had recently received Ara‐C therapy, which is known to cause both central and peripheral neurotoxicity.^29^, ^30^ Three years later, one of these patients developed CNS‐LCH with MRI findings of infundibular thickening and absence of the neurohypophyseal high signal.

Overall, these data suggest that plasma NFL is frequently elevated in adults with histiocytosis‐associated ND, including those undergoing treatment with BRAF and/or MEK inhibitors.

### IL‐1RT2 is a suggested novel diagnostic and therapy‐response candidate biomarker

Next, we assessed the transcriptional expression of our top CSF ND‐LCH markers, including IL‐1RT2, MMP12, and MCP‐3, in a published RNA‐seq dataset by Mass et al.,^28^ which analyzed sorted microglia from mice bearing the *BRAF* ^V600E^ mutation compared to wild‐type controls. In this dataset, IL‐1RT2 and MMP12 showed increased mRNA expression in *BRAF* ^V600E^‐positive microglia relative to controls (Figure 4B), whereas MCP‐3 showed no consistent change.

Since our initial proteomics analyses were performed using the Olink platform, which offers high sensitivity and specificity but is not available for clinical use, we next aimed to validate these top markers using conventional ELISA. This approach provides a more affordable and straightforward option for laboratories worldwide. We performed ELISA to detect IL‐1RT2, MMP12, and MCP‐3 in CSF samples but were unable to detect a signal for either MMP12 or MCP‐3, likely because their concentrations were below the detection limits of the ELISA assays used. In contrast, IL‐1RT2 was readily detectable in the CSF of ND‐LCH patients (Figure 4C).

Importantly, ELISA confirmed elevated IL‐1RT2 levels in a largely independent set of samples: 75% of the samples were new, collected from the same patients at different time points, reflecting distinct clinical scenarios (e.g., changes in treatment, disease progression, ND status). Levels in control CSF samples were substantially lower (range 0–17.6 pg/mL, median 0 pg/mL, mean 5 pg/mL) compared to ND‐LCH samples (range 7.2–2122 pg/mL, median 675.4 pg/mL, mean 426.8 pg/mL) (Figure 4C). Furthermore, IL‐1RT2 CSF levels decreased following treatment (Figure 4D). Specifically, longitudinal patient series revealed (see “Patient case series for IL‐1RT2 validation” in Supporting Information) that IL‐1RT2 decreased during regression of CNS lesions in Patient A and remained stable in Patient B during stable ND‐LCH (Figure 4D). In Patient C, disease progression with new bone lesions and increasing NFL was accompanied by increasing IL‐1RT2, and in Patient D, IL‐1RT2 tracked closely with disease suppression and recurrence during and after dabrafenib therapy. Specifically, Patient D did not show clinical or radiological signs of ND‐LCH at the time of the first sample, yet IL‐1RT2 levels were already elevated. During dabrafenib treatment (samples 2–4), IL‐1RT2 levels normalized (Figure 4D, patient D). After treatment discontinuation, early ND‐LCH changes were detected on MRI, at which point IL‐1RT2 reached its highest level (Figure 4D, patient D). This pattern suggests that IL‐1RT2 elevation reflects clinically relevant changes in disease activity and dynamic response to MAKPi and may even precede detectable neuroradiological alterations.

Together, these results suggest that IL‐1RT2 is a promising candidate biomarker for ND‐LCH, warranting validation in larger cohorts.

## DISCUSSION

In this study, we applied targeted proteomics to unique LCH cohorts and age‐matched normal and inflammatory reference groups, which were methodologically matched and processed under uniform laboratory conditions to identify LCH ND‐specific protein signatures in CSF and plasma. These signatures indicate macrophage activation and cytotoxic immune responses, suggesting interplay between innate and adaptive immunity in the neurodegenerative process. We further identified IL‐1RT2 as a novel candidate biomarker for ND‐LCH and validated its potential in CSF using conventional ELISA.

We observed a strong ND‐LCH‐specific proteomic imprint in CSF samples, which was clearly distinguishable from other groups in unsupervised analyses. This distinct profile provides insight into disease mechanisms and opens new avenues for mechanistic studies. ND‐LCH patients also exhibited a unique plasma signature, with some overlap with the CSF profile. Notably, metalloproteases such as MMP12 and MMP9 were among the most highly upregulated proteins in the CSF of patients with ND‐LCH. These enzymes have been previously shown to be expressed in LCH lesions and LCH cells,^4^, ^31^, ^32^ and are known to play key roles in tissue remodeling, inflammation, and blood–brain barrier integrity.^33^ Altered regulation of their inhibitors may therefore contribute to blood–brain barrier disruption or extracellular matrix remodeling in ND‐LCH. Interestingly, by re‐analyzing previously published in vivo RNA‐seq data from a BRAFV600E microglia mouse model,^28^ we observed increased mRNA expression of MMP12 and IL‐1RT2. This should be interpreted cautiously, as mRNA levels do not necessarily reflect protein abundance, and the model does not fully recapitulate human LCH‐associated ND. The analysis therefore provides only supportive, directional evidence of consistency within a BRAFV600E‐driven microglial context. This is consistent with recent findings that *BRAF* ^V600E^ may identify patients at the highest risk of developing ND‐LCH.^34^, ^35^ Moreover, it is supported by recent evidence showing that microglia in histiocytosis adopt an inflammatory phenotype and contribute to astrocytosis and neuronal loss in murine models.^36^ Furthermore, *BRAF* ^V600E^‐positive iPSC‐derived microglia‐like cells have been shown to induce neuronal damage and increase the release of NFL.^37^

Cytokines and chemokines have been proposed to mediate histiocytosis pathogenesis in blood as well as at different tissue sites.^38^, ^39^, ^40^, ^41^, ^42^, ^43^, ^44^, ^45^, ^46^, ^47^ In CSF, we describe a distinct signature in ND‐LCH, including pro‐inflammatory cytokines and the monocyte chemoattractant MCP‐3, supporting the hypothesis that senescent, pro‐inflammatory cytokine‐producing^48^ circulating myeloid cells enter the brain.^49^ However, in ND‐LCH CSF, we also identified immunoregulatory molecules, such as PDL2, PD1^50^ and soluble receptors (IL‐1RT2, TNF‐R2, and IL‐2RA), which can act as decoy agents, neutralizing and modulating cytokine signaling.^51^ This suggests the possibility that the histiocytosis‐affected brain attempts to protect itself by dampening the neuroinflammation driven by *BRAF* ^V600E^‐positive myeloid cells. Indeed, TNF and IL‐1 signaling have been implicated in LCH pathogenesis,^41^, ^43^, ^52^, ^53^, ^54^ and both were found to be elevated in the blood and brains of LCH mice expressing the *BRAF* ^V600E^ mutation in myeloid cells, compared to wild‐type controls.^49^

Regarding CSF biomarkers, the decoy receptor IL‐1RT2 emerged as the strongest biomarker candidate, capable of discriminating ND‐LCH from all other sample groups, including the MS group, a disease with both inflammatory and neurodegenerative components. We note that the lack of alternative neurodegenerative or inflammatory controls limits assessment of IL‐1RT2 specificity. For example, CSF levels of IL‐1RT2 have been reported to be higher in Alzheimer's disease patients as compared to controls, but still lower than in our untreated ND‐LCH patients.^55^

Like most studies, ours has limitations. Additional studies are needed to assess the specificity of IL‐1RT2 as a marker for ND‐LCH as compared to other degenerative diseases, but for patients with verified LCH, the use of IL‐1RT2 appears very promising. There was an age mismatch between patients and controls, mainly due to the ethical difficulty of obtaining healthy pediatric CSF. In addition, comparisons between ND‐LCH and non‐ND LCH may introduce bias, given the slow and often subclinical onset of ND‐LCH. Further studies on biomarkers in ND‐LCH, possibly with even larger control cohorts, are therefore warranted. A limitation of our study is that we did not use *Z*‐score transformation for NFL, which could be considered in future studies. We used a targeted proteomics platform (Olink) due to its high sensitivity and suitability for low‐volume CSF and plasma samples, and we acknowledge that mass spectrometry‐based approaches may provide complementary or expanded biomarker coverage in future studies. Finally, the ROC AUC of 0.99 for IL‐1RT2 implies a near‐perfect diagnostic accuracy, but these preliminary results require confirmation and may decrease upon independent validation.

Importantly, we successfully validated IL‐1RT2 using conventional ELISA, a method that can be readily implemented in clinical laboratories worldwide. Notably, IL‐1RT2 levels declined following BRAF/MEKi treatment, suggesting its potential utility as a treatment‐response candidate biomarker and warranting further evaluation in larger prospective cohorts, including whether IL‐1RT2 elevation may precede detectable neuroradiological findings as indicated in Patient D (Figure 4D). Although inferior to CSF IL‐1RT2, the recently reported CSF biomarkers NFL^20^, ^23^ and OPN^21^ were significantly elevated in ND‐LCH compared to non‐ND‐LCH, supporting their clinical value.

Due to limited sample availability, plasma analyses were more restricted. Nevertheless, plasma NFL outperformed other markers in distinguishing ND‐LCH among LCH patients. Notably, two patients from the prospective validation cohort in Japan were defined as non‐CNS‐LCH and recently treated with Ara‐C, which may reflect treatment‐related neurotoxicity.^29^, ^30^ Furthermore, plasma NFL was also elevated in adults with various forms of histiocytosis‐associated ND, consistent with the previous report,^25^ even during BRAF/MEKi therapy. The persistence of elevated plasma NFL despite BRAF/MEKi treatment supports the conclusion that current therapies are inadequate to fully control ND in histiocytosis, warranting the development of new therapeutic approaches.

## AUTHOR CONTRIBUTIONS

**Egle Kvedaraite:** Methodology; investigation; data curation; writing—original draft; writing—review and editing; validation. **Magda Lourda:** Investigation; writing—review and editing. **Johannes Bastiaan Munting:** Investigation; writing—review and editing. **Matthias Papo:** Investigation; data curation; writing—review and editing. **Martin Jädersten:** Investigation; data curation; writing—review and editing. **Yoko Shioda:** Investigation; data curation; writing—review and editing. **Kenichi Sakamoto:** Investigation; data curation; writing—review and editing. **Daniel Martín Muñoz:** Investigation; data curation; writing—review and editing. **Panagiotis Andriopoulos:** Investigation; writing—review and editing. **Francesco Pegoraro:** Investigation; data curation; writing—review and editing. **Evaldas Laurencikas:** Investigation; data curation; writing—review and editing. **Ingrid Kühnle:** Investigation; data curation; writing—review and editing. **Joakim Wille:** Investigation; data curation; writing—review and editing. **Leonie Naeije:** Investigation; data curation; writing—review and editing. **Helena Olkinuora:** Investigation; data curation; writing—review and editing. **Indranil Sinha:** Investigation; writing—review and editing. **Daniel W. Hagey:** Investigation; writing—review and editing. **Selma Olsson‐Åkefeldt:** Investigation; data curation; writing—review and editing. **Fleur Cohen‐Aubart:** Investigation; data curation; writing—review and editing. **Gustaf Österlundh:** Investigation; data curation; writing—review and editing. **Mattias Svensson:** Investigation; writing—review and editing. **Ahmed Idbaih:** Investigation; data curation; writing—review and editing. **Fredrik Piehl:** Investigation; data curation; writing—review and editing. **Kaj Blennow:** Investigation; writing—review and editing. **Henrik Zetterberg**: Investigation; writing—review and editing. **Tatiana Greenwood von Bahr:** Investigation; data curation; writing—review and editing. **Désirée Gavhed:** Investigation; data curation; writing—review and editing. **Akira Morimoto:** Investigation; data curation; writing—review and editing. **Julien Haroche:** Methodology; data curation; writing—review and editing. **Jan‐Inge Henter:** Methodology; data curation; writing—original draft; writing—review and editing; validation.

## CONFLICT OF INTEREST STATEMENT

The authors declare no conflicts of interest.

## DISCLOSURES

During the preparation of this work, the authors used ChatGPT (by OpenAI) for minor language editing. After using this tool, the authors reviewed and edited the content as needed and take full responsibility for the content of the published article. Figure 1 was created with BioRender.com.

## ETHICS STATEMENT

The study was approved by the Swedish Ethical Review Authority (2019‐03 956), the local ethics committee Comité de Protection des Personnes d'Ile‐de‐France III (2011‐A00447‐34), and the Institutional Review Board of the National Center for Child Health and Development, Japan (2020‐182).

## FUNDING

The study was supported by the Erik och Edith Fernströms Foundation, Sällskapet barnavård Foundation, The Swedish Childhood Cancer Fund (KP2021‐0006 and KP2024‐0012), The Swedish Cancer Society (Dnr 23 3162 Pj), Dr. Åke Olsson Foundation for hematological research, The Region Stockholm (ALF‐project; FoUI‐998 983), the Histiocytosis Association, and the Erdheim‐Chester Disease Global Alliance.

## Supporting information

Supporting File 1.

## Data Availability

The data that support the findings of this study are available from the corresponding author upon reasonable request.
