# Supplementary material for: Targeted proteomics reveal histiocytosis‐associated neurodegeneration signatures
Source: Hemasphere. 2026 Sep 7;10(9):e70470. doi: 10.1002/hem3.70470 (PMC13548782; doi:10.1002/hem3.70470)
Supplement: Supplementary file 1 [file HEM3-10-e70470-s001.docx]

**Supplemental data**

**Targeted Proteomics Reveal Histiocytosis-Associated**

**Neurodegeneration Signatures**

**Methods**

**Patients and samples: discovery LCH cohort**

Plasma samples and cerebrospinal fluid were collected from LCH patients treated or consulted at Karolinska University Hospital, Stockholm, during a 12-year period starting in 2007. The study has been approved by The Swedish Ethical Review Authority (2019-03956). The discovery cohort (Figures 1–3) included 30 patients with Langerhans Cell Histiocytosis (LCH), comprising 14 with central nervous system involvement (CNS-LCH) and 16 without CNS involvement (non-CNS-LCH). Among the CNS-LCH group, 10 patients were identified as having neurodegenerative LCH (ND-LCH), three had CNS pituitary mass lesions otherwise without neurodegeneration, and one had a spinal cord lesion. ND status was not available for the spinal cord lesion case; however, this patient’s CSF proteomic profile clustered with those of ND-LCH patients in later analyses (Figure 2A), and the sample was included in the ND-LCH cluster for all analyses. CSF samples were available from all CNS-LCH patients, including follow-up samples from six of the 11 patients analyzed in the ND-LCH group (including the spinal cord lesion case, from whom only one sample with no follow-up was available), and from 11 non-CNS-LCH patients, one of whom also had a follow-up sample. This resulted in 45 CSF samples: 30 from ND-LCH, three from CNS-LCH without ND, and 12 from non-CNS-LCH. Plasma samples were obtained from 17 patients: four with ND-LCH (including three follow-up samples), one with CNS-LCH without ND (including one follow-up sample), and 12 with non-CNS-LCH. For a correlation analysis between plasma NFL and CSF ND-LCH markers, 16 matched plasma and CSF samples were obtained from 11 LCH patients within a seven-day window (i.e., plasma and CSF samples from each patient were collected within a week): four with ND-LCH (including follow-up samples from two), one with CNS-LCH without ND, and seven with non-CNS-LCH (Figure 3C). Detailed clinical information is provided in Table 1 and Figure 1.

**Patients and samples: controls for the discovery LCH cohort**

For controls, the CSF cohort included non-CNS-LCH patients (mentioned above) as non-neurodegenerative references, alongside 30 anonymized pediatric ALL patients without CNS involvement and 12 healthy young adults (HC; female/male 50%/50%, ages 24–30, mean age 26.2 years), from whom both CSF and plasma were collected. CSF and plasma samples from MS patients (female/male 50%/50%, ages 22–29, mean age 26.2 years) were included as inflammatory controls. For plasma, non-CNS-LCH patients also served as non-neurodegenerative references, complemented by six healthy children recruited at the Pediatric Day Surgery Unit, Karolinska University Hospital, as additional non-neurodegenerative references (female/male 33%/66%, ages 3-10, mean age 5.7 years).

**Patients and samples: prospective validation cohorts**

For the first validation cohort, prospective plasma samples were collected from histiocytosis patients at the National Reference Center for Histiocytosis, Paris, France, during a period of 2.7 years starting in 2019. This part of the study was approved by the local ethics committee [Comité de Protection des Personnes d’Ile de France III (#2011-A00447–34)] and was conducted in accordance with the Declaration of Helsinki. Initially, 60 patients were recruited. One patient with suspected LCH but no formal histological diagnosis was excluded from the analyses, resulting in 10 patients with neurodegeneration (ND), and 49 patients without neurodegeneration (non-ND). All patients were sampled at least once, and two patients with ND were sampled twice, resulting in analysis of 12 ND samples and 49 non-ND samples analyzed (Figure 4A). Detailed clinical information is provided in Supplementary Table 1.

For the second validation cohort, prospective plasma samples were collected from LCH patients at the Center of Child Health and Development, Japan, during a period of 0.7 years starting in 2020. This research has been approved by the Institutional review board of the National Center for Child Health and Development (ethical approval number: 2020-182). In total, 15 patients were recruited: six patients with ND-LCH (with or without DI), three LCH patients with diabetes insipidus (DI) (CNS-LCH without ND), and six LCH patients without CNS-LCH (non-CNS) at the time of the sampling. All patients were sampled at least once, and one ND-LCH patient was sampled twice, resulting in seven ND-LCH, three DI, and six non-CNS samples analyzed (Supplemental Figure 1). Detailed clinical information is provided in Supplementary Table 2.

**IL-1RT2 validation using ELISA**

To validate the IL-1RT2 enzyme-linked immunosorbent assay (ELISA), we analyzed 11 CSF control samples from 11 ALL patients without CNS involvement and 21 CSF samples from five ND-LCH patients recruited at Karolinska University Hospital, Stockholm (Figure 4C). Of the ND-LCH samples, four longitudinal samples were collected from four individual patients, and five samples were obtained from one patient. A total of 15 out of 21 ND-LCH samples (71.4%) were newly collected, while six samples (28.6%) were previously analyzed using the Olink platform. For four of the five ND-LCH patients, CSF samples were available both before and after BRAF and/or MEK inhibitors (Figure 4D). In three of the four patients there was an off-treatment (any treatment) sample available that was compared to subsequent samples on/after treatment (any treatment) (Figure 4D). Clinical course descriptions of the four patients are provided below, with corresponding IL-1RT2 ELISA levels in relation to BRAF and/or MEK inhibitors or other treatment shown in Figure 4D.

**Patient case series for IL-1RT2 validation**

Patient A is a male diagnosed with LCH at the age of 25 years. Detailed clinical characteristics are provided in Table 1 of a previously published study by Sveijer et al (<https://doi.org/10.1111/bjh.18247>), where this patient corresponds to patient 9. Briefly, the maximal extent of disease was multisystem risk organ (RO)-, with skin and CNS involvement, including DI and possible clinical and radiological ND-LCH with subjective cognitive symptoms of neurodegeneration as well as neuroradiological abnormalities. The patient had CNS-risk lesions. Sample 1 was collected when the patient was 39 years old while the patient was not receiving any active treatment (timepoint 0 in Figure 4D; the IL-1RT2 level was 142 pg/mL). Sample 2, with an IL-1RT2 level of 124 pg/mL, was obtained following four cycles of cladribine combined with dexamethasone as part of treatment evaluation; however, there was no clear clinical improvement, and MRI indicated slight progression of CNS lesions. Sample 3 was collected after initiation of cobimetinib therapy, at which point MRI showed clear regression of CNS lesions and a reduction in NFL levels. Sample 4 was collected while cobimetinib treatment was still ongoing, and the IL-1RT2 level had decreased to 95 pg/mL.

Patient B is a male diagnosed with LCH at the age of 6 months. Detailed clinical characteristics are provided in Table 1 of a previously published study by Henter et al (<https://doi.org/10.1111/bjh.17781>), where this patient corresponds to patient 1. Briefly, the maximal extent of disease was multisystem RO-, with bone, probably skin, and CNS involvement, including DI and clinical and radiological ND-LCH with balance problems and typical neuroradiological findings of ND-LCH. The patient had CNS-risk lesions. At sample 1 (IL-1RT2 level was 925 pg/mL), the patient was on cytarabine and oral dexamethasone. The first treatment-free sample was obtained at age 6.4 years (timepoint 0 in Figure 4D); the IL-1RT2 level was then 302 pg/mL. Between the second and third samples, dabrafenib was tried twice but discontinued due to side effects. At sample 3, when IL-1RT2 levels had decreased to 101 pg/mL, the patient was not on treatment but had 1 month earlier discontinued dabrafenib due to side effects. At sample 4, the patient had been on trametinib for 18.6 months, and the IL-1RT2 level was very low (31 pg/mL).

Patient C is a female diagnosed with LCH at the age of 23 years. Detailed clinical characteristics are provided in Table 1 of a previously published study by Sveijer et al (<https://doi.org/10.1111/bjh.18247>), where this patient corresponds to patient 8. Briefly, the maximal extent of disease was multisystem RO-, with skin and CNS involvement, including DI and clinical and radiological ND-LCH with ataxia, nystagmus, need of support for walking, as well as neuroradiological abnormalities. The patient did not have CNS-risk lesions. At sample 1 the patient was 23.1 years old and was untreated (timepoint 0 in Figure 4D), and the IL-1RT2 level was markedly elevated to 2122 pg/mL. Prior to sample 2, with an IL-1RT2 level of 410 pg/mL, the patient received as initial treatment 3 cycles of cytarabine without effect followed by vemurafenib with partial response but side effects that prompted a switch to trametinib which led to complete PET-response and partial reversal of her long-standing DI, however, her neurological symptoms did not improve. Due to side effects of trametinib, she received 6 cycles of cladribine with stable clinical symptoms but she suffered repeated episodes of pancreatitis. Next, the patient was treated with clofarabine plus trametinib, followed by trametinib plus dabrafenib. Directly prior to sample 3 she had been off treatment for approximately four months and during this untreated period the disease progressed with new bone lesions and increasing NFL, and the IL-1RT2 level had also increased (804 pg/mL). Prior to sample 4, the patient had been on methotrexate and 6-mercaptopurine for about four months, following several targeted therapies including trametinib plus dabrafenib, binimetinib with encorafenib, and binimetinib monotherapy, each discontinued after one to two months due to side effects.

Patient D is a male diagnosed with LCH at the age of 32 months. Detailed clinical characteristics are provided in Table 1 of a previously published study by Henter et al (<https://doi.org/10.1111/bjh.17781>), where this patient corresponds to patient 2. Briefly, the maximal extent of disease was multisystem RO-, with bone, skin, possibly lung, and CNS involvement, including DI and radiological ND-LCH. The patient had CNS-risk lesions. At sample 1 the patient was 3.2 years old and was on vinblastine/prednisolone/6-MP (LCH-IV protocol, stratum I) (timepoint 0 in Figure 4D). Notably, at this time, the patient did not show clinical or radiological signs of ND-LCH, yet the IL-1RT2 level was already elevated (318 pg/mL). At sample 2, the patient had been on dabrafenib for approximately three months. At sample 3, the patient had been on dabrafenib for about six months. At sample 4, the patient had completed around one year of dabrafenib treatment, which was then discontinued due to clinical stability; the IL-1RT2 level was then 7 pg/mL. Thus, during dabrafenib treatment (samples 2–4), IL-1RT2 levels normalized and remained normal. At sample 5, when the IL-1RT2 level had increased (589 pg/mL), the patient had been off targeted treatment for approximately one year, was receiving oral 6-MP/methotrexate, and was showing signs of early neurodegenerative CNS disease; dabrafenib was reinitiated shortly afterwards, leading to neuroradiological ND-LCH improvement after 15 months on dabrafenib.

**CSF and plasma sampling**

The CSF was collected through a standard lumbar puncture procedure, followed by centrifugation at 4°C and collection of the supernatant into small aliquots that were then stored at -80°C to preserve CSF integrity for subsequent analysis. Plasma samples were collected in whole-blood tubes containing EDTA (ethylenediaminetetraacetic acid), aliquoted, and stored at -80°C to ensure sample integrity. For LCH patients (including non-ND-LCH, CNS-LCH without ND, and ND-LCH groups) and healthy pediatric controls, peripheral blood was diluted 1:1 in phosphate buffered saline (PBS), plasma collected following Ficoll separation, and frozen under the same storage conditions as described above. All CSF and plasma samples were processed within two hours of collection.

**Quantification of soluble factors**

Targeted proteomics was performed by quantifying selected soluble proteins across all samples from all cohorts using the proximity extension assay (PEA) (Olink AB, Uppsala, Sweden). Each sample was analyzed with two panels: Immuno-Oncology (v.3111) and Cardiovascular III (v.6113), each targeting 92 proteins, resulting in the measurement of 184 proteins in total. One sample, a follow-up CSF sample from an LCH patient with CNS-LCH without ND, was excluded due to a quality control (QC) warning. Additionally, one protein, MCP-1, was present in both panels, while six proteins (IL1a, IL2, IL5, CXCL12, IL4, and IL13) had most of their data (up to 75% in LCH plasma and even more in CSF) falling below the lower detection limit. These six proteins were excluded from the analyses, resulting in a final total of 177 Olink analytes available for further investigation. For the remaining analytes, values below the limit of detection were retained as reported in the Olink NPX output without imputation.

NFL levels in CSF were measured using an in house ELISA with a normal reference value of <380 ng/L (<https://pubmed.ncbi.nlm.nih.gov/29370869/>). As plasma NFL levels are substantially lower than in CSF, plasma NFL levels were quantified using the more sensitive Quanterix Simoa NFL assay, which has a dynamic range of 1.9 to 1812 pg/mL. Age-specific normal reference values for plasma NFL are as follows: 7 pg/mL for individuals aged 5 to <18 years, <10 pg/mL for 18 to <51 years, <15 pg/mL for 51 to <61 years, <20 pg/mL for 61 to 70 years, and <35 pg/mL for individuals aged 70 years or older (<https://pubmed.ncbi.nlm.nih.gov/35865350/>). Results were adjusted to account for sample dilutions. All NFL analyses were conducted at the Clinical Neurochemistry Laboratory, Sahlgrenska University Hospital, Mölndal, Sweden.

For the validation using ELISA, IL-1RT2 levels in CSF were measured at Karolinska Institute using the human IL-1RT2 solid-phase sandwich ELISA from Invitrogen (cat #: EHIL1R2). Briefly, 21 CSF samples from the five LCH patients described above and 11 control samples (ALL patients without CNS involvement sampled once) were diluted 2-fold in Diluent A and plated in duplicates (100μl/well) in a pre-coated IL-1RT2 plate. The samples were incubated for 2.5 hours at room temperature, followed by washing and subsequent processing according to the manufacturer’s instructions. Results were adjusted to account for sample dilution.

**Olink and NFL data analyses**

In total, 177 Olink analytes, presented as normalized protein expression (NPX) values (Olink Proteomics’ arbitrary unit on a log2 scale), were analyzed alongside absolute NFL levels, which were log2-transformed for consistency with the Olink data. All statistical analyses, including the Wilcoxon rank-sum test and false discovery rate (FDR) adjustment, were conducted using base R functions. Dimensionality reduction was performed on all CSF samples, comprising a total of 178 analytes (177 Olink and NFL), by first calculating principal components and then using the five most significant components to compute uniform manifold approximation and projection (UMAP) coordinates with a minimum distance parameter of 0.2, primarily for exploring the data structure and visualization purposes ^25^. Differential protein expression in CSF between ND-LCH patients and non-ND controls (including non-CNS-LCH, CNS-LCH without ND, ALL, and HC), as well as between ND-LCH and MS patients, was assessed using the non-parametric Wilcoxon rank-sum test. P-values were adjusted for multiple comparisons using the FDR method. A volcano plot was generated to visualize the results, with the x-axis representing log2 fold changes and the y-axis displaying the -log10 FDR-adjusted p-values. The significance thresholds were set at |log2(fold change)| ≥ 0.5 and an FDR-adjusted p-value < 0.05. Statistical significance was indicated by a dashed line at a y-axis value of 1.3, corresponding to an FDR-adjusted p-value of 0.05. For the MS comparison, a non-linear transformation was applied to the x-axis to enhance the visualization of fold changes across a wide range. The ggplot2 and ggrepel packages were used to generate the dotplots and volcano plots, with ggplot2 for creating the plots and ggrepel for labeling significant results. Heatmaps were generated with the ComplexHeatmap, circlize, and RColorBrewer packages. Proteins were ranked by FDR-adjusted p-value for CSF and p-value for plasma with and without adjustment in order to avoid false negatives as matched plasma sample size was limited; the top 10 proteins (ND-LCH vs. non-ND CSF in Figure 2C), all significant proteins (ND-LCH vs. non-ND plasma in Figure 3A) and top 30 proteins (ND-LCH vs. MS CSF in Figure 2D) were selected for visualization in heatmaps. Data were scaled using Z-scores, and hierarchical clustering was performed using the complete linkage method and Euclidean distance metric. Receiver operating characteristic (ROC) analyses were performed using the rocit package in R (empirical method), with ND-LCH defined as the positive class and all other samples as the negative reference. The optimal threshold was determined using the Youden Index performed on the same dataset (Figure 2F, 3F). To assess the overlap of significantly altered proteins Venn diagrams were created using the VennDiagram R package with scaled circle sizes to reflect the number of proteins unique to or shared between the two sample types (Figure 3B).

Spearman correlation analysis between plasma NFL and CSF ND-LCH markers were calculated between plasma NFL (log₂-transformed) and the top 30 CSF ND-LCH markers, defined by highest positive fold change and FDR-adjusted P < 0.05 from differential expression analysis, as well as OPN (Figure 3C). The analysis was performed using 16 matched LCH samples, where CSF and plasma were collected within 7 days. Correlation coefficients and corresponding P-values were computed using the cor() and cor.test() functions in R. Results were visualized as a heatmap using the pheatmap package, with statistical significance indicated (*P < 0.05, **P < 0.01, ***P < 0.001). Scatter plots with linear regression lines were generated using ggplot2. All analyses were performed in R (v4.3.0) using the following packages: dplyr, ComplexHeatmap, RColorBrewer, circlize, corrplot, ggplot2, and pheatmap.

Statistical analyses among multiple groups were performed using Kruskal-Wallis with Dunn’s test (Figure 2E, 3D, 4A), between two groups Mann-Whitney test (Figure 3E, 4A), and mixed-effects model with subject as a random effect was used to compare patients and controls (Figure 4C) using GraphPad Prism (version 10.0.3).

**Supplemental Figures**

**Supplemental Figure 1. Plasma NFL in the Japanese cohort.** Plasma NFL levels in LCH patients (n = 15) from Japanese cohort (mean with SEM shown, arrows in dark pink point to patients that have recently received Ara-C treatment).

**Supplemental Tables**

**Supplemental Table 1. Clinical characteristics of the prospective histiocytosis cohort from France.**

|  |  | ND | non-ND |
| --- | --- | --- | --- |
| Patient number | | 10 | 49 |
| Diagnosis | |  |  |
|  | ECD | 5 (50) | 29 (59) |
|  | LCH | 0 (0) | 13 (27) |
|  | ECD-LCH | 5 (50) | 3 (6) |
|  | RDD | 0 (0) | 2 (4) |
|  | *ECD or RDD | 0 (0) | 1 (2) |
|  | *ECD or LCH | 0 (0) | 1 (2) |
| Sex Female/Male | | 5/5 (50/50) | 18/31 (37/63) |
| Age at sampling, min-max (median) | | 41-77 (56.5) | 22-90 (63) |
| Molecular findings | |  |  |
|  | *BRAF*^V600E^ | 9 (90) | 19 (39) |
|  | *BRAF*^V600E^ + *JAK2* | 1 (10) | 1 (2) |
|  | *MAP2K1* | 0 (0) | 7 (14) |
|  | *KRAS* | 0 (0) | 1 (2) |
|  | **Other | 0 (0) | 2 (4) |
|  | None | 0 (0) | 19 (39) |
| Treatment at sampling | |  |  |
|  | BRAFi | 3 (30) | 10 (20) |
|  | MEKi | 1 (10) | 16 (33) |
|  | BRAFi+MEKi | 5 (50) | 1 (2) |
|  | PEG‑IFNα | 1 (10) | 6 (12) |
|  | IL-1Ra | 0 (0) | 3 (6) |
|  | None | 0 (0) | 13 (27) |
| Organ involvement since diagnosis | |  |  |
|  | Bone | 9 (90) | 42 (86) |
|  | Perirenal/periadrenal | 5 (50) | 23 (47) |
|  | Lung | 1 (10) | 19 (39) |
|  | Large vessel | 6 (60) | 17 (35) |
|  | Heart | 3 (30) | 14 (29) |
|  | Facial/orbit | 5 (50) | 13 (27) |
|  | Hypothalamic/Pituitary | 3 (30) | 12 (24) |
|  | Skin/xanthelasma | 3 (30) | 12 (24) |
|  | CNS | 10 (100) | 10 (20) |
|  | Mesenteric | 0 (0) | 5 (10) |
|  | Lymph nodes | 0 (0) | 2 (4) |
|  | Retroperitoneal | 0 (0) | 1 (2) |
|  | Pleura | 0 (0) | 1 (2) |
|  | Gastric | 0 (0) | 1 (2) |
|  | Peritoneal | 0 (0) | 1 (2) |
|  | Gut | 0 (0) | 1 (2) |
|  | Hepatobiliary tract | 0 (0) | 1 (2) |
|  | Breast | 0 (0) | 1 (2) |

**Notes:**

Data are presented as number of patients with percentage in parentheses, unless otherwise indicated. Age at sampling is reported in years as range with median in parentheses.

*Diagnosis pending between the two listed entities.

**"Other" includes one patient with BRAF c.1457_1471del (p.Val486_Gly490del) and one patient with DDX41 mutation, both in the non-ND group.

**Abbreviations:**

BRAFi = BRAF inhibitor; CNS = central nervous system; ECD = Erdheim-Chester disease; IL‑1Ra = interleukin‑1 receptor antagonist; LCH = Langerhans cell histiocytosis; MEKi = MEK inhibitor; ND = neurodegenerative; PEG‑IFNα = pegylated interferon-α2a; RDD = Rosai-Dorfman disease.

**Supplemental Table 2. Clinical characteristics of the prospective LCH cohort from Japan.**

|  |  | ND-LCH | DI | non-CNS |
| --- | --- | --- | --- | --- |
| Patient number | | 6 | 3 | 6 |
| Sex Female/Male | | 4/2 (67/33) | 0/3 (0/100) | 1/5 (17/83) |
| Age at sampling, min-max (median) | | 3.4-23.1 (16.3) | 9.1-43.8 (42.3) | 0.8-18.11 (8.6) |
| Molecular findings | |  |  |  |
|  | *BRAF*^V600E^ | 3 (50) | 0 (0) | 2 (33) |
|  | No mutation found | 1 (17) | 0 (0) | 0 (0) |
|  | Not assessed | 2 (33) | 3 (100) | 4 (67) |
| Treatment at sampling | |  |  |  |
|  | Ara-C | 0 (0) | 0 (0) | 2 (33) |
|  | Systemic steroids | 3 (50) | 0 (0) | 2 (33) |
|  | Vincristine | 0 (0) | 0 (0) | 2 (33) |
|  | Methotrexate | 0 (0) | 0 (0) | 2 (33) |
|  | Hydroxyurea | 0 (0) | 1 (33) | 1 (17) |
|  | IVIG | 6 (100) | 0 (0) | 0 (0) |
|  | None | 0 (0) | 2 (67) | 3 (50) |

**Notes:**

Data are presented as number of patients with percentage in parentheses, unless otherwise indicated. Age at sampling is reported in years as range with median in parentheses.

**Abbreviations:**

Ara-C = cytosine arabinoside (Cytarabine), DI = diabetes insipidus, IVIG = intravenous immunoglobulin therapy, ND-LCH = neurodegenerative Langerhans cell histiocytosis, non-CNS = Langerhans cell histiocytosis patients without central nervous system involvement.
